# Stereotactic and Robotic Minimally Invasive Thermal Ablation of Malignant Liver Tumors: A Systematic Review and Meta-Analysis

**DOI:** 10.3389/fonc.2021.713685

**Published:** 2021-09-23

**Authors:** Pascale Tinguely, Iwan Paolucci, Simeon J. S. Ruiter, Stefan Weber, Koert P. de Jong, Daniel Candinas, Jacob Freedman, Jennie Engstrand

**Affiliations:** ^1^ Division of Surgery, Department of Clinical Sciences, Karolinska Institutet at Danderyd Hospital, Stockholm, Sweden; ^2^ Department of Visceral Surgery and Medicine, Inselspital, Bern University Hospital, University of Bern, Bern, Switzerland; ^3^ ARTORG Center for Biomedical Engineering Research, University of Bern, Bern, Switzerland; ^4^ Department of Hepato-Pancreato-Biliary Surgery and Liver Transplantation, University Medical Center Groningen, University of Groningen, Groningen, Netherlands

**Keywords:** liver neoplasms, ablation techniques, stereotaxic techniques, computer-assisted therapies, minimally invasive surgical procedures

## Abstract

**Background:**

Stereotactic navigation techniques aim to enhance treatment precision and safety in minimally invasive thermal ablation of liver tumors. We qualitatively reviewed and quantitatively summarized the available literature on procedural and clinical outcomes after stereotactic navigated ablation of malignant liver tumors.

**Methods:**

A systematic literature search was performed on procedural and clinical outcomes when using stereotactic or robotic navigation for laparoscopic or percutaneous thermal ablation. The online databases Medline, Embase, and Cochrane Library were searched. Endpoints included targeting accuracy, procedural efficiency, and treatment efficacy outcomes. Meta-analysis including subgroup analyses was performed.

**Results:**

Thirty-four studies (two randomized controlled trials, three prospective cohort studies, 29 case series) were qualitatively analyzed, and 22 studies were included for meta-analysis. Weighted average lateral targeting error was 3.7 mm (CI 3.2, 4.2), with all four comparative studies showing enhanced targeting accuracy compared to free-hand targeting. Weighted average overall complications, major complications, and mortality were 11.4% (6.7, 16.1), 3.4% (2.1, 5.1), and 0.8% (0.5, 1.3). Pooled estimates of primary technique efficacy were 94% (89, 97) if assessed at 1–6 weeks and 90% (87, 93) if assessed at 6–12 weeks post ablation, with remaining between-study heterogeneity. Primary technique efficacy was significantly enhanced in stereotactic vs. free-hand targeting, with odds ratio (OR) of 1.9 (1.2, 3.2) (n = 6 studies).

**Conclusions:**

Advances in stereotactic navigation technologies allow highly precise and safe tumor targeting, leading to enhanced primary treatment efficacy. The use of varying definitions and terminology of safety and efficacy limits comparability among studies, highlighting the crucial need for further standardization of follow-up definitions.

## Introduction

Thermal ablation therapy is a validated curative-intended treatment option for malignant liver tumors, mainly hepatocellular carcinoma (HCC) and liver metastases from colorectal cancer (CRLM) (1, 2). Following encouraging oncological outcome results for small tumors in the setting of limited disease, thermal ablation has been introduced into international treatment guidelines for both HCC and CRLM (3, 4).

The key advantage of ablative treatments is their tissue-sparing nature, allowing preservation of a maximum of functioning liver tissue and favouring combination with a minimally invasive treatment access to further reduce procedure-related morbidity. The main challenge in achieving treatment success in such minimally invasive environments is the accurate and safe positioning of ablation probes to acquire adequate ablation volumes with full tumor coverage (5, 6). The use of (contrast-enhanced) ultrasound (US) imaging allows dynamic intraoperative tumor visualization and instrument guidance both in surgical and interventional radiology settings (7–9). For lesions remaining invisible due to small size, deep central location, obstructing gas/ribs, or changes in liver parenchyma (10), computed tomography (CT) or magnetic resonance (MR) guidance enhances visibility but introduces constraints due to radiation exposure (11) or procedure-related complexity. Especially for tumors requiring complex targeting trajectories, such as in the liver dome or caudal lobe, a safe and efficient targeting is often precluded (12, 13).

To improve tumor accessibility, targeting accuracy, and treatment safety, stereotactic navigation systems have been introduced for use in minimally invasive surgery. These aim to enhance treatment precision by integrating computer assistance with imaging data, allowing stereotactic guidance of surgical instruments (14). Over the last two decades, increasing clinical experience with commercially available navigation devices has been reported, including several summary articles highlighting their main advantages (15–21). However, no systematic review of the available literature on the clinical application of this technology exists, and the true impact on procedural and clinical outcomes remains unknown. The aim of this study was to critically review and quantitatively summarize the available literature on targeting accuracy, procedural efficiency, and treatment efficacy when using stereotactic or robotic navigation technology for thermal ablation of malignant liver tumors in a minimally invasive setting.

## Methods

### Search Strategy

This study was conducted following the Preferred Reporting Items for Systematic reviews and Meta-Analyses (PRISMA) guidelines (22). The PRISMA checklist is available as **Supplementary File 1**. A systematic literature review was performed on May 20, 2020, searching the online databases Medline, Embase, and the Cochrane Central Register of Controlled Trials for all available full-text articles. Search terms were organized according to the population, intervention, comparison, and outcomes (PICO) criteria, without including comparison or outcome criteria to keep the search as broad as possible. Given the expected paucity of published literature, no limitation of publication dates was applied. The complete search strategy is shown in **Table 1**.

**Table 1:** Search strategy.

Specification	Population		Intervention (Part 1)		Intervention (Part 2)		Comparison/Outcome
	*Patients with liver tumors*		*Stereotactic*		*Thermal ablation*		*Accuracy, efficiency, efficacy*
**Text word search (Medline and Embase)**	liver:tiab	AND	stereota*:tiab	AND	ablation:tiab	AND	Not applied
	hepatic:tiab		navigat*:tiab				
			robot*:tiab				
**Thesaurus search MeSH (Medline, Cochrane Library)**	liver neoplasms		computer-assisted therapy		ablation techniques		
			stereotaxic techniques		radiofrequency ablation		
**Thesaurus search Emtree (Embase)**	liver cancer/exp		stereotactic treatment/exp		ablation therapy/exp		
	liver metastasis/exp		robot assisted surgery/exp				

Boolean Operators “OR” applied between search terms (rows) within PICO columns and “AND” between PICO columns.:tiab, Title and abstract./exp, explosion search.

### Study Selection

Eligible studies included original articles on adults, reporting on procedural or clinical outcomes when using stereotactic or robotic guidance for targeting and thermal ablation of malignant liver tumors, and using a minimally invasive (laparoscopic or percutaneous) approach. Stereotactic or robotic guidance was defined as the utilization of a tracked ablation probe or aiming device for targeting and subsequent therapeutic thermal ablation. Included studies reported on one or more of the following outcomes related to stereotactic thermal ablation of liver tumors: i) targeting accuracy, ii) procedural efficiency and safety, and iii) treatment efficacy. Excluded were i) review articles, ii) conference abstracts, iii) studies focusing on image fusion without the use of a tracked ablation probe or aiming device for tumor targeting, iv) studies analyzing efficacy of combined transarterial chemoembolization (TACE) and stereotactic ablation of liver tumors, v) case reports defined as <10 patients treated with navigated/robotic ablation, and vi) full texts written in languages other than English.

Two authors (PT and JE) independently determined eligibility for each citation by sequential review of titles, abstracts, and full texts using the predefined criteria. In case of disagreements, consensus was reached by group discussion. Reference lists from included studies were reviewed for additional citations not identified by the original search. Covidence (23) and Mendeley were used for screening and reference citation management.

### Data Extraction and Risk of Bias Assessment

All data extracted for systematic review and meta-analysis were entered into a data spreadsheet available as [**Supplementary File 2**]. Main outcomes were i) targeting accuracy, defined as targeting errors resulting after stereotactic ablation probe positioning; ii) procedural efficiency and safety, including duration of the overall procedure and of stereotactic ablation probe positioning, numbers of probe readjustments, radiation exposure reported as dose length product (DLP), hospital length of stay (LOS), and clinical complications; and iii) treatment efficacy, including rates of technical success, primary and secondary technique efficacy, and local tumor progression (LTP). Definitions of treatment efficacy were summarized using the standardized terminology and reporting criteria for image-guided tumor ablation (24). The authors’ detailed descriptive definitions of all endpoints are available in the [**Supplementary File 2**].

For quantitative analyses, data from studies with overlapping patient populations were obtained from the most relevant publications. These were chosen by prioritizing studies with the largest sample size and then by studies published most recently. Risk of bias in individual studies was evaluated for all outcomes according to the ROBINS-I tool (25), and studies with high risk of bias were excluded from meta-analysis. Studies reporting on ablation for uncommon subpopulations of liver tumors (i.e., very large tumors) were considered *high risk of selection bias* toward safety and efficacy outcomes. Comparative observational studies reporting outcomes without matching of tumors with respect to targeting complexity (e.g., tumor location) were considered *medium risk of bias due to confounding*. No quantitative analyses were performed for efficiency outcomes (probe readjustments, procedure, and targeting durations/DLP) and LTP, since heterogeneity across studies in definition and assessment of these outcomes was deemed too important.

### Data Synthesis and Meta-Analysis

Baseline characteristics were summarized, and weighted averages, rates, and odds ratios (ORs) were reported according to individual number of lesions or patients, as appropriate. Pooled estimates were calculated based on a modified inverse variance method, applying a continuous/binary random effects model given the assumed heterogeneity of included studies (DerSimionian–Laird method) (26). To avoid skewing of the variance toward zero, an arcsine transformation was applied for outcomes with small reported proportions (major complications, mortality) (27). Subgroup meta-analysis was performed to account for known sources of heterogeneity due to differing definitions for complication rates and primary technique efficacy. Between-study heterogeneity was reported as I^2^ statistic and chi-square test of homogeneity. Sensitivity analyses were performed by comparing results from subgroup analyses vs. overall results and random-effects vs. fixed-effects analyses. OpenMetaAnalyst (28), R (R Core Team, 2019), and RStudio (RStudio Inc., USA) were used for meta-analysis and generation of graphics.

## Results

**Figure 1:**
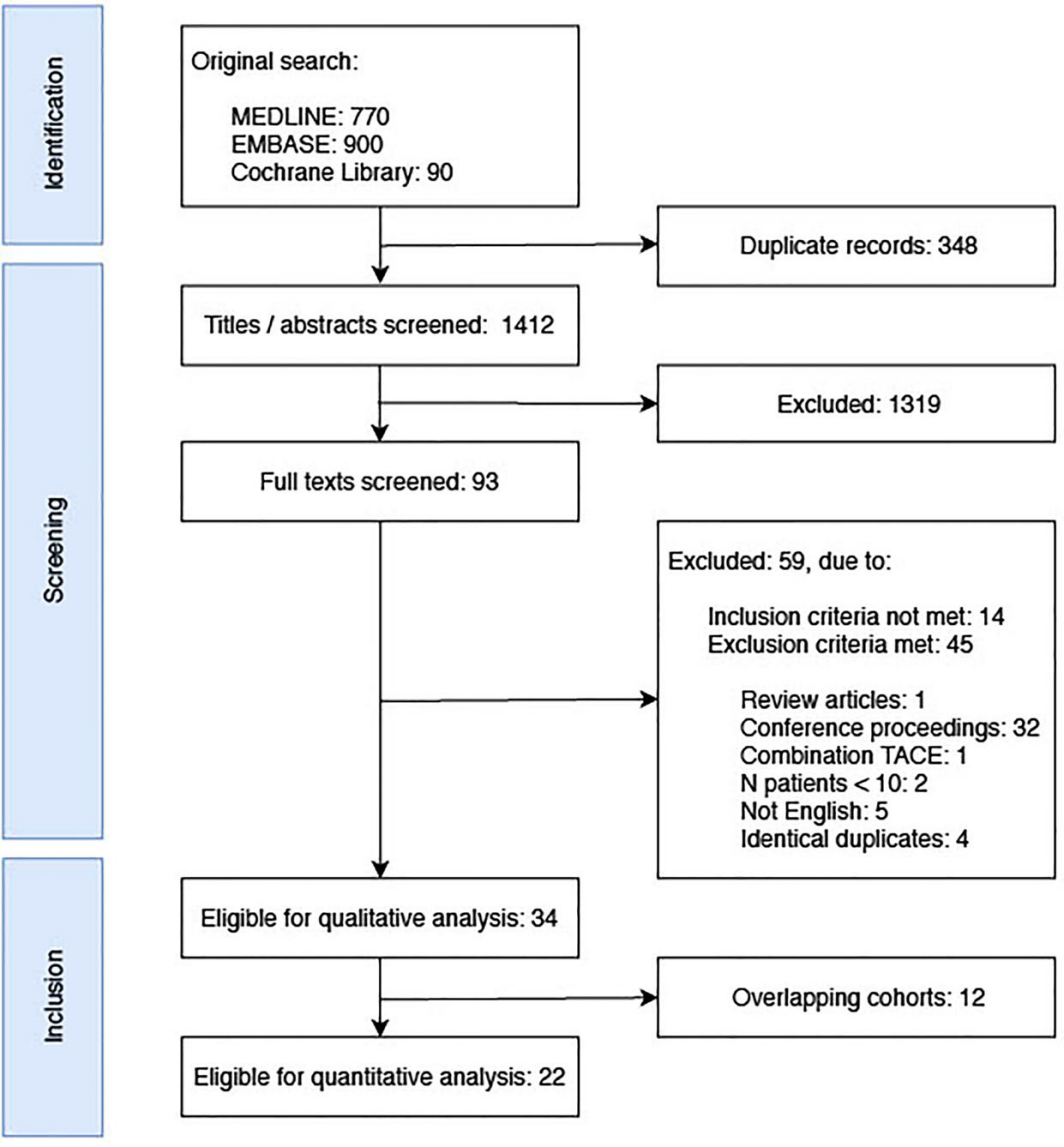
Flowchart of study selection after systematic literature review.

After screening a total of 1,412 articles, 93 original works published in English and reporting on a minimum of 10 patients treated with stereotactic or robotic thermal ablation for malignant liver tumors were included for full-text screening. After additional exclusion of 59 works, 34 articles were included for qualitative analysis and 22 for quantitative analysis (**Figure 1**).

All of the 34 included works were single-center studies, of which 26 were retrospective studies (29–54), three were prospective case series (55–57), three were prospective cohort studies (58–60), and two were randomized controlled trials (61, 62). Two studies reported results using a laparoscopic treatment access (36, 55); the other 32 reporting on thermal ablations using a percutaneous approach. Six studies applied robotic targeting using mechanical tracking (39–41, 44, 60, 62), seven an electromagnetic (EM)-tracked dynamic technique (34, 43, 51, 55, 57, 59, 61), and the other 21 an optically tracked stereotactic aiming device. Baseline study, patient and lesion characteristics, applied ablation and navigation techniques, and reported outcomes of the included works are shown in **Table 2**. All data extracted from included studies are available in the **Supplementary File 2**. Results from meta-analyses including sensitivity analyses and selection bias assessment are available in **Supplementary File 3**.

**Table 2:** Baseline characteristics of included studies.

Study, year published	Study design	Tumor entity				Lesion subgroup	N patients	N lesions	Lesion size [mm] °	Ablation technique	Imaging technique	Tracking technology	Navigation device	Anesthesia Ventilation	Outcomes reported and data overlap/risk of bias ^#^			
		HCC	Metas	CCC											Acc	Eff^#^	Saf	Eff
TinguelyA, 2020 (29)	R	1	1				153	301	15	MWA	CT	Optical	CAS-ONE^a^	General Jet-Vent	1	1 **^#^**	1	1
SchullianA, 2020 (30)	R	1	1			CT-invisible	60	199	HCC: 29 Metas: 25	RFA	CT	Optical	Stealth Station Treon plus^b^	General Tube discon			1 **^#^**	1 **^#^**
Schaible, 2020 * (41)	R	1	1	1			not defined	249	18.8	MWA	CT	Mechanical	MAXIO^c^	General Tube discon		1 **^#^**	1	1
SchullianB, 2020 (48)	R	1				Previous resection	34	140	30	RFA	CT	Optical	Stealth Station Treon plus^b^	General Tube discon			1 **^#^**	1 **^#^**
SchullianC, 2020 (49)	R	1	1	1		Size >8 cm	34	41	90	RFA	CT	Optical	Stealth Station Treon plus^b^	General Tube discon			1 **^#^**	1 **^#^**
SchullianD, 2020 (50)	R	1	1			Multiple n ≥4	92	549	2.7	RFA	CT	Optical	Stealth Station Treon plus^b^	General Tube discon		1 **^#^**	1	1
Volpi, 2019 (51)	R	1	1			Not visible on US	21	27	12	RFA/MWA	CT	EM	IMACTIS^d^	General Jet-Vent	1	1 **^#^**	1	1
SchullianE 2019 (52)	R	1	1	1		Octogenarians	36	70	27	RFA	CT	Optical	Stealth Station Treon plus^b^	General Tube discon			1 **^#^**	1 **^#^**
SchullianF, 2019 (53)	R	1				Caudate lobe	20	24	15	RFA	CT	Optical	Stealth Station Treon plus^b^	General Tube discon			1 **^#^**	1 **^#^**
Perrodin, 2019 (54)	R		Non- CRLM				23	40	13.5	MWA	CT	Optical	CAS-ONE^a^	General Jet-Vent			1 **^#^**	1
SchullianG 2019 (31)	R	1	1	1		Hepatic dome	177	238	22	RFA	CT	Optical	Stealth Station Treon plus^b^	General Tube discon			1 **^#^**	1 **^#^**
SchullianH 2019 (32)	R	1				Subcardiac	79	114	25	MWA	CT	Optical	Stealth Station Treon plus^b^	General Tube discon			1 **^#^**	1 **^#^**
ZhangA, 2019 (61)	RCT	1	1			Solitary nodule ≤5 cm	20	20	24	RFA/MWA	CT	EM	IG4^e^	Local/sedation		1 **^#^**	1	1
Lachenmayer, 2019 (33)	R	1					88	174	16	MWA	CT	Optical	CAS-ONE^a^	General Jet-Vent	1 **^#^**	1 **^#^**	1 **^#^**	1
Heerink, 2019* (62)	RCT	1	1 + Benign			N ≤3 ≤5 cm	18	47	21.2	MWA	CT	Mechanical	Needle Positioning System^f^	General Tube discon	1	1 **^#^**	1	1
BeyerA, 2018 (58)	P	1	1				18	18	20.6	MWA	CT	Optical	CAS-ONE^a^	General Tube discon	1	1 **^#^**	1	1
ZhangB, 2018 (34)	R	1				N = 1 3–8 cm	19	19	41.4	MWA	CT/US	EM	Fusion image navigation system^g^	General		1 **^#^**	1	1
BaleA, 2017 (35)	R		Mamma CA				26	64	2.8	RFA	CT	Optical	Stealth Station Treon plus^b^	General Tube discon			1 **^#^**	1 **^#^**
TinguelyB, 2017 (36)	R	1	1				51	346	16.7	MWA	Preoperative CT	Optical	CAS-ONE^a^	General Tube discon		1 **^#^**	1	1
Hirooka, 2017 (59)	P cohort study	1				N = 1 ≤5 cm	27	27	23.9	Bipolar RFA	CT/US	EM	3D sim-Navigator^h^	General			1	1
Engstrand, 2016 (37)	R	1	1			Invisible on US N ≤ 2 ≤ 3cm	17	25	14.9	MWA	CT	Optical	CAS-ONE^a^	General Jet-Vent	1	1 **^#^**	1	
BaleB, 2016 (38)	R		Melanoma			≤5 cm	20	75	17	RFA	CT	Optical	Stealth Station Treon plus^b^	General Tube discon			1 **^#^**	1 **^#^**
BeyerB, 2016 * (39)	R	1	1				not defined	34	19.1	MWA	CT	Mechanical	MAXIO^c^	General Apnea	1	1 **^#^**	1	1
AbdullahA, 2015 * (40)	R	1	1				20	40	23	RFA/MWA	CT	Mechanical	MAXIO^c^	General Tube discon		1 **^#^**	1	1
Mbalisike, 2014 * (60)	P cohort study	1	1	1		N = 1 Prior TACE (3 months)	30	30	23.2	MWA	CT	Mechanical	MAXIO^c^	Local/sedation	1	1 **^#^**	1	
Sindram, 2014 (55)	P	1	1				13	34	18.0	MWA	US	EM	AIM Guidance System^i^	General		1 **^#^**	1	1
SchullianI, 2014 (42)	R	1	1	1		Prior laparoscopic liver packing	47	120	24	RFA	CT	Optical	Stealth Station Treon plus^b^	General Tube discon		1 **^#^**	1 **^#^**	1 **^#^**
Mauri, 2014 (43)	R	1	1			Not/incompletely visible on US/CEUS	175	295	13	RFA/MWA	US-CT US-MRI	EM	VirtuTrax^j^	General Tube discon/end-exp	1		1	1
Abdullah,B 2013 * (44)	R	1	1				11	17	21.8	RFA	CT	Mechanical	ROBIO EX^c^	General Tube discon		1 **^#^**	1	1
BaleC, 2011 (45)	R		1				63	189	20	RFA	CT	Optical	Stealth Station Treon plus^b^	General Tube discon		1 **^#^**	1 **^#^**	
Haidu, 2011 (46)	R			1			11	36	3	RFA	CT	Optical	Stealth Station Treon plus^b^	General Tube discon		1 **^#^**	1 **^#^**	
WidmannA, 2011 (47)	R	1	1	1		N ≤ 7 ≤12 cm	90	177	29	RFA	CT	Optical	Stealth Station Treon plus^b^	General Tube discon			1	1
WidmannB, 2011 (56)	P	not defined					20	35	31	RFA	CT	Optical	Stealth Station Treon plus^b^	General Tube discon	1		1	
Liu, 2011 (57)	P	1				US: invisible CT/MRI: yes ≤ 3 nodules	18	18	18.5	MWA	US-CT US-MRI	EM	MyLab90 System, Virtual Navigator^k^	Local/sedation		1 **^#^**	1	1
Total		28	25		8		1,531	3,806										

°Median or average, as reported.

*Robotic guidance. Median or average, as reported. ^#^Excluded from meta-analysis.

^a^CAScination AG, Switzerland; ^b^Medtronic, USA; ^c^Perfint Healthcare, India; ^d^Imactis, France; ^e^Veran Medical Technologies Inc., USA; ^f^DEMCON Advanced Mechatronics, Netherlands; ^g^Self-developed; ^h^Hitachi Healthcare, Japan; ^i^InnerOptic Technology Inc., USA; ^j^CIVCO Medical Solutions, USA; ^k^Esaote SpA, Italy.

RCT, randomized controlled trial; R, retrospective; P, prospective; HCC, hepatocellular carcinoma; CRLM, colorectal cancer liver metastases; CCC, cholangiocellular carcinoma; CEUS, contrast-enhanced ultrasound; TACE, transarterial chemoembolization; RFA, radiofrequency ablation; MWA, microwave ablation; DLP, dose length product; EM, electromagnetic.

**Figure 2:**
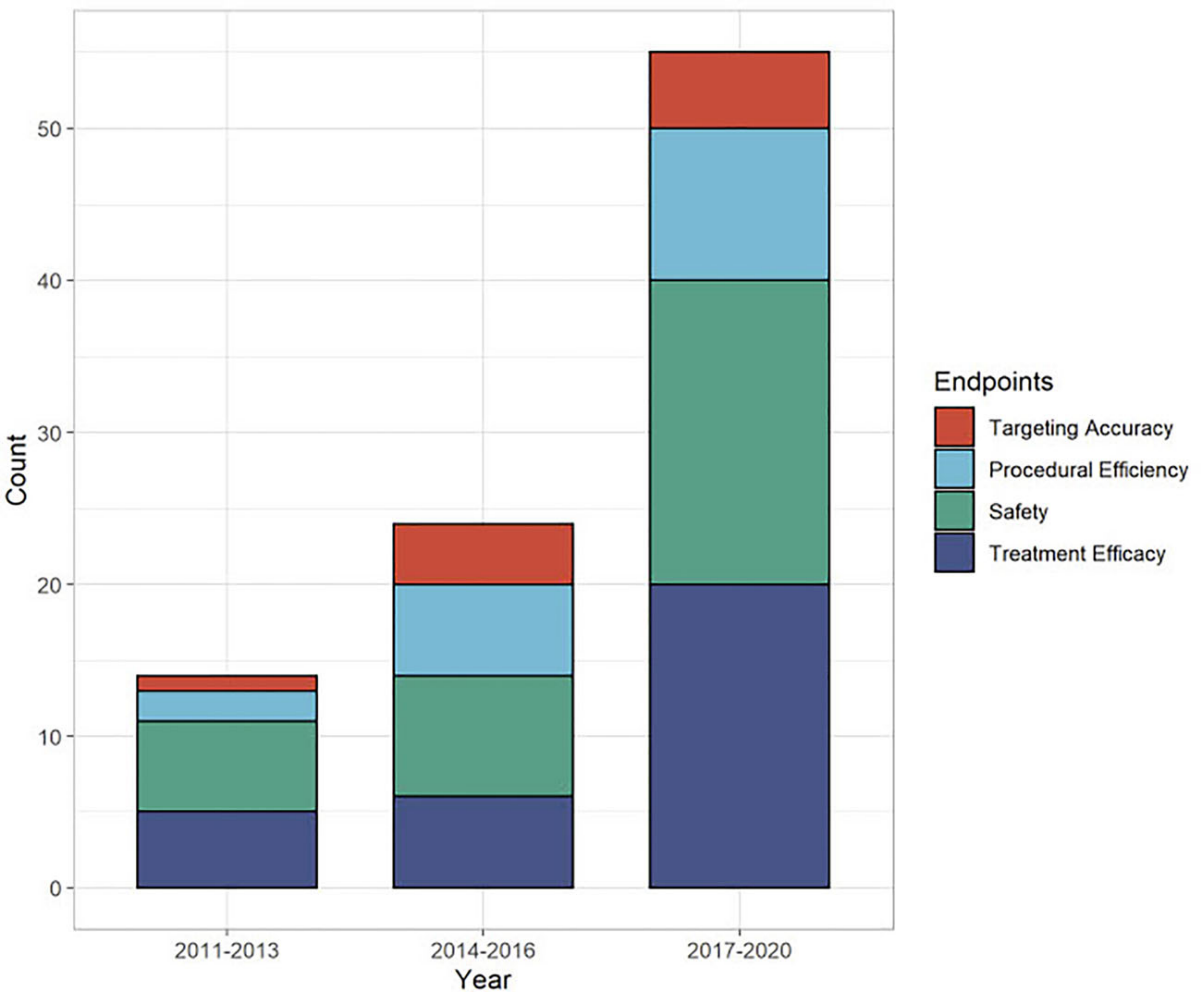
Distribution of reported endpoints over time (n = 34 studies).

The published literature on stereotactic or robotic guidance for thermal ablation of liver tumors increased continuously since the first clinical series in 2011. This was the case regarding all reported endpoints and most prominently for safety and treatment efficacy, as illustrated in **Figure 2**.

### Targeting Accuracy

**Figure 3:**
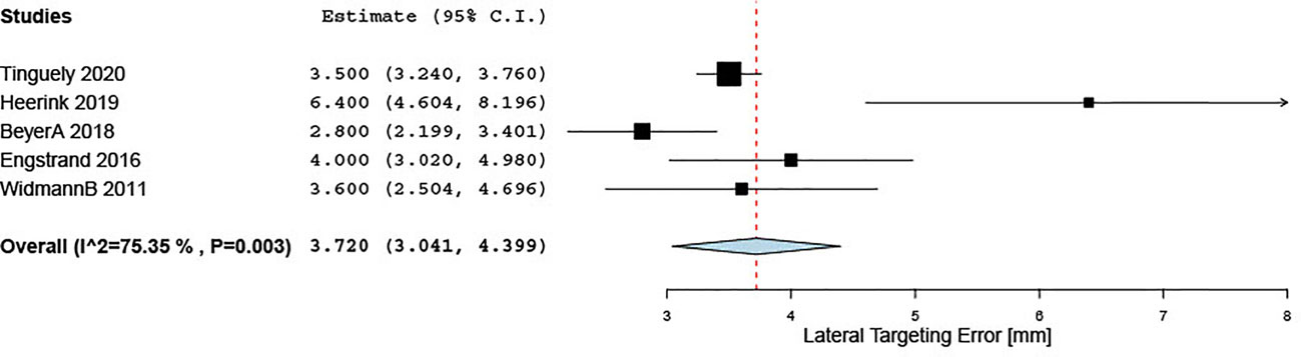
Forest plot for pooled estimate of lateral targeting error.

Of the 34 included studies, 10 reported on targeting accuracy, of which nine reported Euclidean, lateral, or angular targeting errors after stereotactic ablation probe positioning. The remaining study reported a 95.6% targeting accuracy, defined as the center of the ablated zone being located within a 5-mm range from the preoperatively defined ideal target point, as assessed on a 24-h CT/MRI scan (43). Pooled estimates for Euclidean, lateral, and angular targeting errors were 5.3 (95% CI 3.9, 6.7), 3.7 (3.0, 4.4), and 2.4 (1.7, 3.1) mm, respectively, with significant between-study heterogeneity (**Table 3**). Summary estimates for lateral targeting errors are displayed in **Figure 3**.

**Table 3:** Summary of targeting accuracy.

Study, year published	Euclidean error [mm]	Lateral error [mm]	Angular error [°]
TinguelyA, 2020 (29)		2.9 ± 2.3	2.0 ± 1.2
Volpi, 2019 (51)	22 ± 19°		
Heerink, 2019 * (62)	10.2 ± 5.2	6.4 ± 4.2	4.5 ± 3.2
BeyerA, 2018 (58)	3.7 ± 1.2	2.8 ± 1.3	2.7 ± 1.5
Engstrand, 2016 (37)	5.8 ± 3.2	4.0 ± 2.5	2.7 ± 2.9
BeyerB, 2015 (39) * **^#^**	3.1 ± 2.5		
Mbalisike, 2014 (60) * **^#^**	5.3 ± 1.8		
WidmannB, 2011 (56)		3.6 ± 2.5	1.3 ± 1.2
Pooled estimates (95% CI)	5.3 (3.9, 6.7)	3.7 (3.0, 4.4)	2.4 (1.7, 3.1)
Heterogeneity (I^2, p-value)	(92.6%, <0.001)	(75.3%, 0.003)	(83.9%, <0.001)

* Robotic guidance.

^#^ Only accuracies of initial probe placements are shown (before eventual re-adjustments).

Only accuracies of initial probe placements are shown (before eventual readjustments).

° Defined as “first-pass control”: aiming at approximately 1 cm from the tumor. Not included in weighted average.

Three studies investigated factors influencing targeting accuracy, of which two studies performed univariable between-group analyses and one group used multivariable linear regression. Mauri et al. (43) showed no influence on “correct targeting” by tumor entity, lesion characteristics, and applied guidance and ablation techniques. Widmann et al. (56) found larger lateral errors at the ablation probe tip in lesions located in “subphrenic plus fat” as opposed to “clear parenchymal” positions and in Segment II vs. Segment IV. Tinguely et al. (29) reported statistically significant higher lateral targeting errors with raising targeting trajectory lengths (0.2 mm per additional cm) and when targeting tumors in cirrhotic livers (by 0.7 mm) in a multivariable model, with no influence of challenging lesion locations or complex targeting trajectories on accuracy.

### Procedural Efficiency and Safety

Eighteen studies reported on procedural efficiency, and all included works reported on safety related to stereotactic targeting for thermal ablation. The need for readjustment of ablation probes due to insufficient accuracy was reported varyingly across studies. Four studies reported mean numbers of ablation probe readjustments of n = 0 (62), 0.8 (40), 1.1 (60), and 2.4 (61). Nine authors reported relative numbers of probe readjustments per patient [4.8% (51), 5.6% (58), 35% (44), and 60% (40)] or per lesion [1% (29), 4% (37), 5.6% (58), 8.8% (55), and 41.2% (39)]. Mean overall procedure duration ranged between 18.3 and 254.5 min in 12 studies and navigated targeting duration between 1.5 and 36.3 min in eight reporting studies. Mean total DLP ranged between 807 and 2,216 mGy * cm in 11 studies reporting overall radiation exposure. Hospital length of stay ranged between 0 and 7 days in 18 reporting studies.

**Figure 4:**
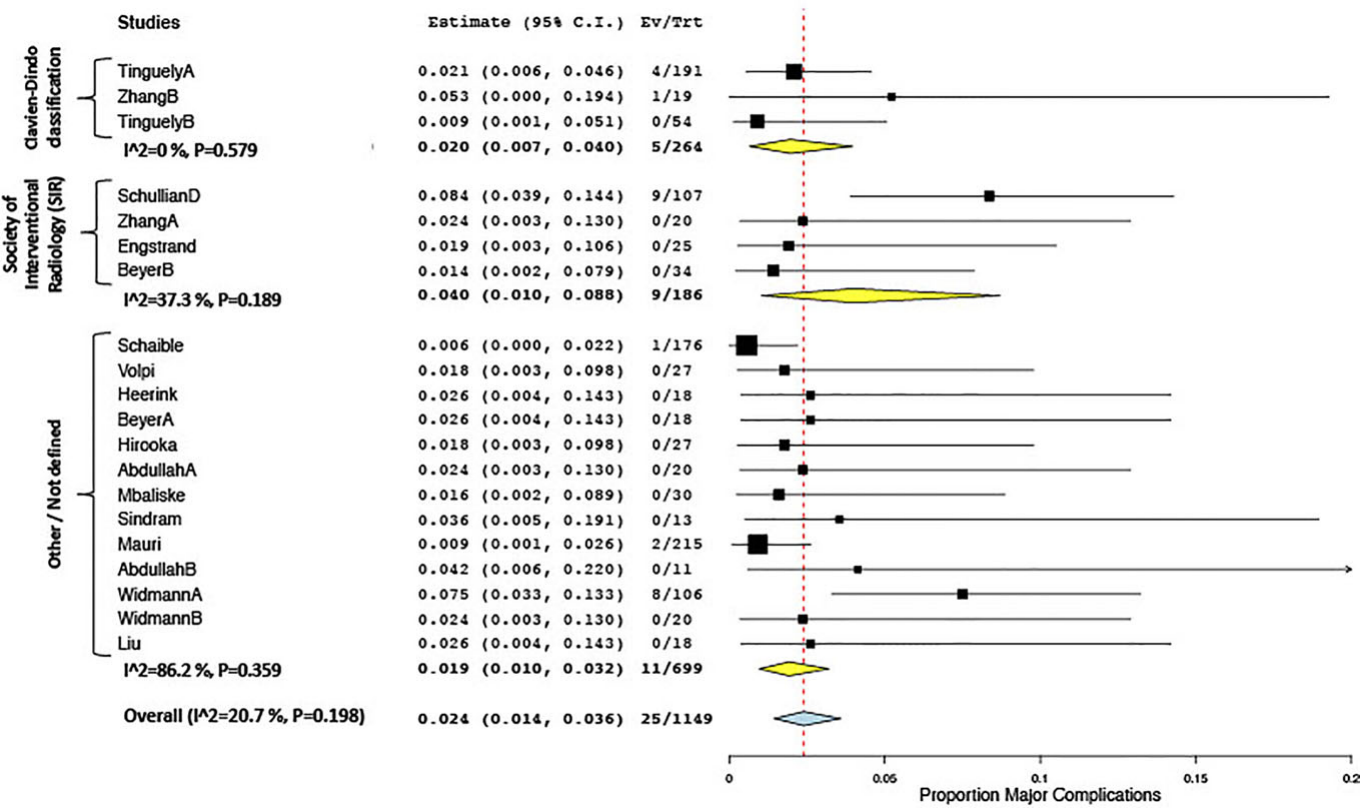
Forest plot for pooled estimates of major complication rates according to definitions of morbidity.

All included studies reported on treatment-related complications, applying varying types of definitions. Twelve studies used the definitions proposed by the Society of Interventional Radiology (SIR) (63), eight applied the Clavien–Dindo classification (64), two applied the definitions proposed by Ahmed et al. (24), one applied the CIRSE classification, and 11 studies used other definitions or did not further specify. The overall complication rate ranged between 0% and 57.9%, the pooled estimate being 11.4% (CI 6.7, 16.1; I^2^ 87.9%, p < 0.01) in 16 studies included for meta-analysis. Major complications ranged between 0% and 20.5%, the 20.5% rate being reported in a study of patients undergoing stereotactic radiofrequency ablation (RFA) for very large (≥8 cm) tumors (49). The overall pooled estimate for major complication rate was 2.4% (CI 1.4, 3.6; I^2^ 20.7%, p = 0.198), which was lower in the subgroup applying the Clavien–Dindo classification (2.0%; CI 0.7, 4.0) vs. the subgroup applying the SIR classification (4.0%; CI 1.0, 8.8) (**Figure 4**). Mortality rates ranged between 0% and 4.3%, with a pooled estimate of 0.8% (CI 0.4, 1.4; I^2^ 0%, p = 0.99) in 20 studies included for meta-analysis.

### Treatment Efficacy

**Figure 5:**
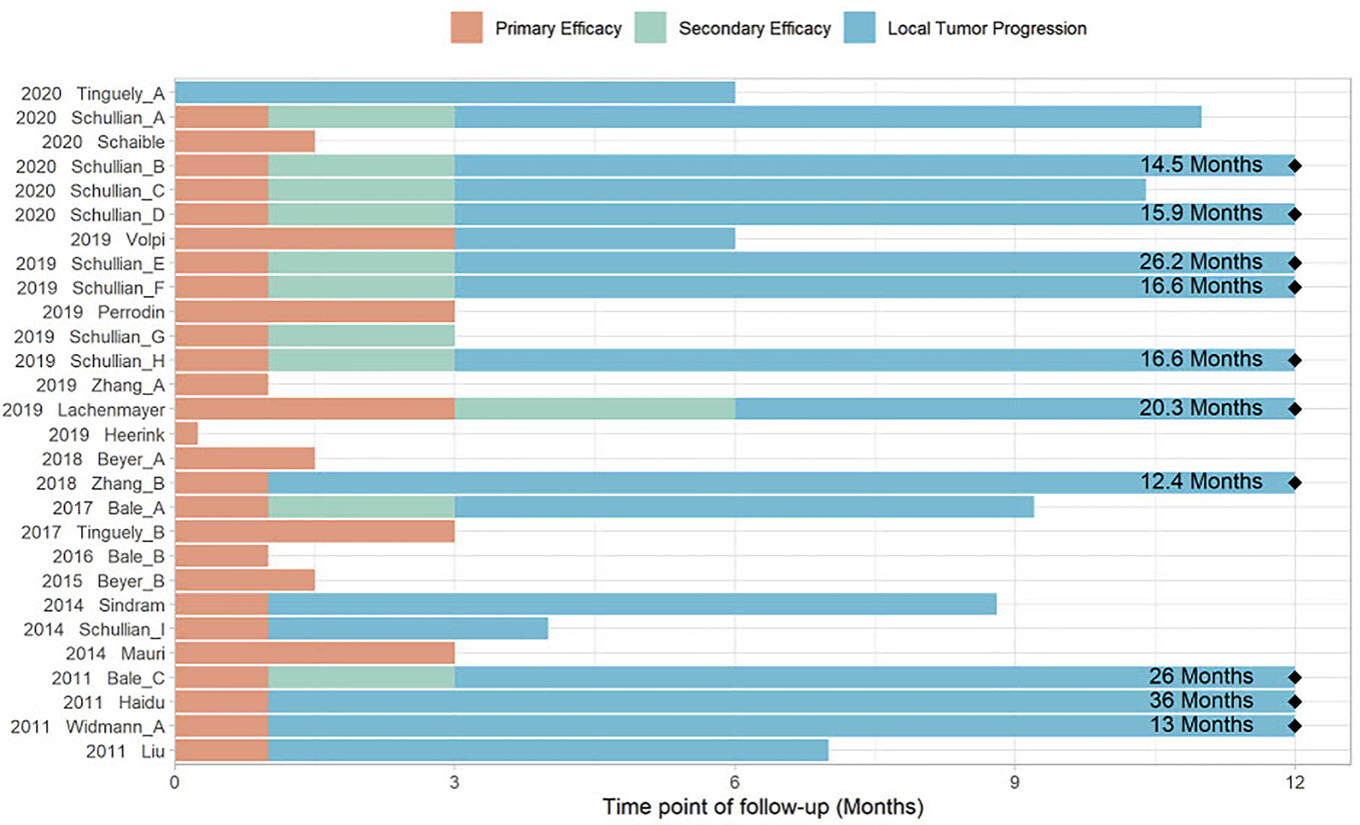
Definitions and time points of reported follow-up assessments.

Thirty-one of the 34 included studies reported on treatment efficacy. Varying definitions were reported for all treatment efficacy outcomes, including the time points for assessment and the duration of follow-up. Six studies (29, 33, 34, 41, 46, 61) referred to the terminology for follow-up assessment after ablation of liver tumors proposed by Ahmed et al. (24), while 13 studies applied similar definitions without explicitly referencing this classification. Individual descriptions and time points of follow-up assessments are available for each study in the **Supplementary File 2**. Varying time points and durations of follow-up assessments in studies reporting treatment efficacy are illustrated in **Figure 5**.

**Figure 6:**
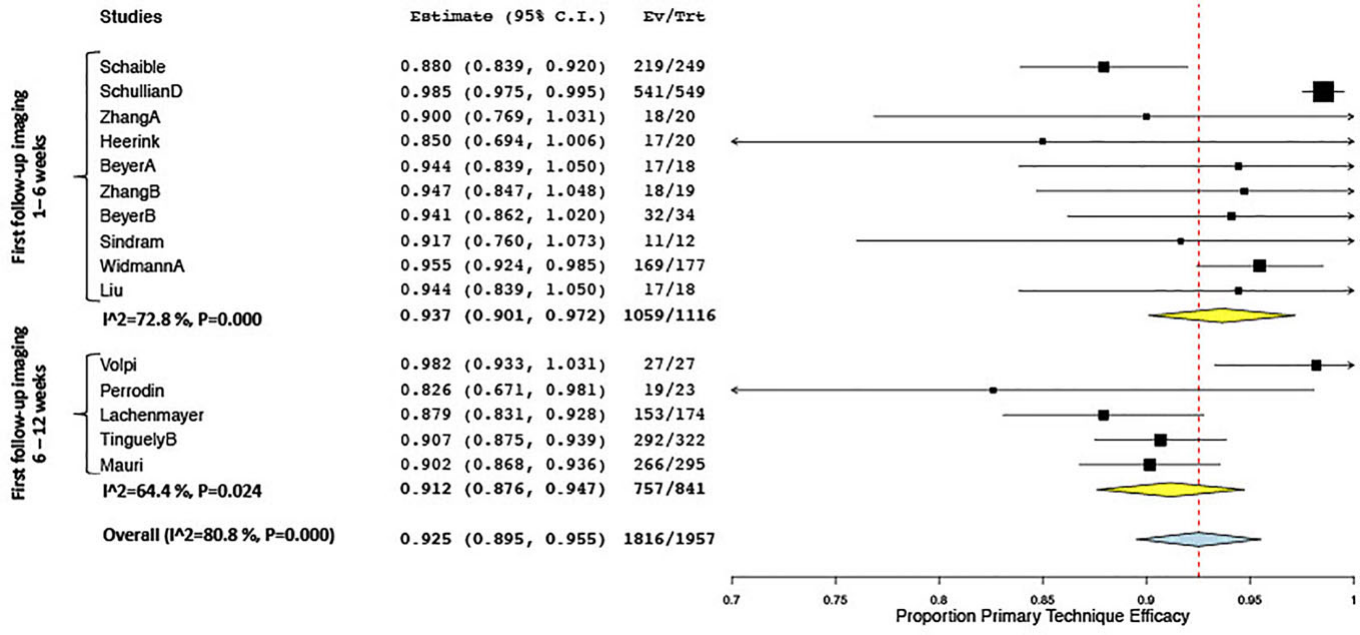
Forest plot of pooled odds ratio of primary technique efficacy after stereotactic vs. free-hand targeting.

Keeping in mind this variability in definitions as well as the varying specific inclusion criteria for patients and lesions in several studies [e.g., very large tumors ≥8 cm (49), vanishing lesions (30)], reported rates for technical success ranged from 90.2% to 100% (18 studies), for primary technique efficacy from 80.5% to 100% (27 studies) and for secondary technique efficacy from 90.2% to 100% efficacy (13 studies), and for LTP from 0% to 54% (21 studies). Quantitative analysis of primary technique efficacy (i.e., complete tumor ablation at the first follow-up imaging) according to time points of the first follow-up is summarized in **Figure 3**. Primary technique efficacy rates were reported to be higher in studies performing a first follow-up after 1–6 weeks (pooled estimate 93.6%; CI 88.9, 97.1) than in studies assessing primary technique efficacy at 6–12 weeks (pooled estimate 90.1%; CI 87.2, 92.7). Despite subgroup analysis, a statistically significant between-study heterogeneity remained in the former group (**Figure 6**).

Six studies presented analyses on factors influencing treatment efficacy when using stereotactic tumor targeting, four of which included multivariable regression analyses. Tinguely et al. (29) showed a statistically significant influence of tumor size (</>3 cm) and targeting accuracy (</>5 mm) on LTP, with no influence by more complex intrahepatic tumor locations, in a multivariable model accounting for clustering by using Generalized Estimating Equation (GEE). Schaible et al. (41) reported tumor size (</>3 cm) next to the type of targeting approach (stereotactic vs. free-hand) to be a significant predictor of primary technique efficacy in a similar logistic GEE model. Lachenmayer et al. (33) found vessel proximity and tumor size (</>3 cm) as independent predictors for LTP. Hirooka et al. (59) reported the free-hand as opposed to the stereotactic approach to be significantly associated with “local residual recurrence” in Cox regression analysis. In univariable between-group comparisons, Widmann et al. (47) reported differences in “technique effectiveness” for lesion size </>5 cm and hollow viscera vicinity and no differences for tumor entity and lesion location. Bale et al. (45) found differences in “local recurrence” rates for lesions in proximity to vessels, bile ducts, and hollow organs.

### Comparative Studies

Nine studies compared stereotactic vs. “free-hand” ablation for varying endpoints, including two randomized controlled studies (61, 62) and three prospective cohort studies (59, 60) of which one used matched-pair analysis (58). Main study characteristics and reported results for targeting accuracy, procedural efficiency, and safety and treatment efficacy are summarized in **Table 4**.

**Table 4:** Comparison of stereotactic vs. free-hand ablation targeting for thermal ablation.

Study	Study design	Matching method	N patients (lesions) stereotactic cohort	Ablation technique	Main outcomes (stereotactic vs. control group)								
					Targeting errors	N probe readjustments	N skin punctures	Overall procedure time	Targeting time	Total DLP	Complications	Primary technique efficacy	Local tumor progression
Schaible 2020 (41)*	R	None (Regression analysis)	? (246)	MWA							ns (↓)	↑	
ZhangA, 2019	RCT	n/a	20 (20)	RFA MWA		↓	ns (↓)		ns (↑)	↓		ns (↑)	
Heerink, 2019 (62)*	RCT	n/a	31 (47)	MWA	↓	↓		ns (↑)	↑	↑		ns (↓)	
BeyerA, 2018 (58)	P (R manual cohort)	Matched pairs	18 (18)	MWA	ns (↓)			ns (↓)		↓		ns (↑)	
ZhangB, 2018 (34)	R	None	19 (19)	MWA			↑		↓		ns (↑)	ns (↑) ↑ first session	ns (↓)
Hirooka, 2017 (59)	P cohort study	None	27 (27)	Bipolar RFA									ns (↓)
BeyerB, 2015 *	R	None	? (34)	MWA	ns (↓) ↓ after manual correction			↓		↓		ns (↓)	
AbdullahA, 2015 (40)*	R	None	20 (40)	RFA MWA						ns (↑)			
Mbalisike, 2014*	P cohort study	None	30 (30)	MWA	↓	↓		↑	↓	↓			

*Robotic guidance. RCT, randomized controlled trial; R, retrospective; P, prospective; HCC, hepatocellular carcinoma; CRCLM, colorectal cancer liver metastases; CCC, cholangiocellular carcinoma; RFA, radiofrequency ablation; MWA, microwave ablation; DLP, dose length product; ns nonsignificant. ↑↓ statistically significant differences, (↑↓) statistically non-significant differences.

Targeting accuracy was shown to be significantly enhanced when using stereotactic targeting in three out of four studies [one of them after manual adaptation of the ablation probe (39)]. The randomized controlled trial by Heerink at al (62). confirmed enhanced accuracy specifically for out-of-plane trajectories (5.9 vs. 10.1 mm) and showed a significant reduction of ablation probe repositionings in robotic vs. free-hand ablations (0 *vs.* 1, primary study endpoint). This was confirmed by Zhang et al. (61) showing fewer instrument readjustments (2.4 vs. 4.95) when using EM-guided targeting and by Mbalisike et al. (60) when using robotic as opposed to conventional CT guidance (1.1 *vs.* 3 readjustments).

Durations for overall procedures and for ablation probe positionings were reported variably across studies. Zhang et al. (61) showed a significant reduction in the number of CT scans used for interventions (7 vs. 10), in CT fluoroscopy time, and in total DLP when using EM-guided as opposed to free-hand targeting. Four other studies showed a reduction, and two studies showed an increase in total DLP in the stereotactic vs. free-hand cohort using (**Table 2**). In the RCT of Heerink et al. (62), the number of navigational CT scans tended to be lower in stereotactic vs. free-hand procedures (5 vs. 7), and the increase in DLP was presumably due to the larger scan field necessary to include the optical reference fiducials.

**Figure 7:**
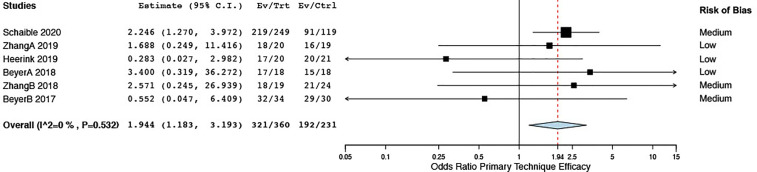
Forest plot for pooled estimates of primary technique efficacy rates according to time points of first follow-up.

With respect to treatment efficacy, Zhang et al. (34) showed a higher complete ablation rate in the first session (3 days after ablation) when using stereotactic EM guidance but equal primary technique efficacy at 1 month. All six studies included for meta-analysis assessed primary technique efficacy at a first follow-up imaging between 1 and 6 weeks. Stereotactic and robotic guidance led to enhanced primary technique efficacy rates as opposed to free-hand targeting, with a pooled OR of 1.94 (CI 1.18, 3.19) (**Figure 7**). Sensitivity analyses comparing random- to fixed-effects analysis showed comparable results, and the risk of publication bias was nonsignificant for this result (Egger’s test: t = -1.24, p = 0.2840) (**Supplementary File 3**).

Four propensity score matched analyses compared stereotactic thermal ablation for different subtypes of patients or lesions. Stereotactic RFA yielded similar safety and treatment efficacy outcomes when comparing CT “invisible” tumors ablated with MR image fusion to CT-visible tumors (30), octogenarians to a younger study population (52), lesions in a subphrenic location to non-dome locations (31), and HCC in a subcardiac position to a non-subcardiac location (32).

## Discussion

This work is a summary of the currently published knowledge on procedural and clinical benefits of applying minimally invasive stereotactic and robotic navigation technology for thermal ablation of malignant liver tumors. To our knowledge, this is the first systematic review and meta-analysis on this topic.

Stereotactic navigation technologies for use in liver surgery aim to enhance precision when performing tissue-sparing treatments such as “atypical” resections not following standard anatomical landmarks or locally targeted interventions such as thermal ablations. For the latter case specifically, the aims are to i) *ensure adequate oncological results* in a minimally invasive setting by precise ablation probe positioning and ii) *enhance safety* by defining trajectories that avoid injury to critical anatomical structures. Stereotactic navigation systems enable quantification of the accuracy with which ablation probes are positioned by measuring the error between the planned “optimal” and the final “real” probe position. This facilitates the intraoperative decision regarding an eventual probe readjustment and, importantly, allows a reproducible and comparable evaluation of targeting success. As summarized herein, pooled estimates for targeting accuracy in over 650 targeted lesions ranged from 2.4 to 5.3 mm, with relatively small confidence intervals and with all comparative studies showing reduced targeting errors as compared to free-hand techniques (**Table 4**).

The pooled estimates for major complication rates for different definitions ranged between 1.8% and 5.2%, as expected when applying tissue-sparing treatments in a minimally invasive setting. Importantly, the number of probe readjustments was significantly reduced in three comparative studies, including two randomized controlled trials. This decrease in liver punctures until reaching adequate probe positions toward n = 1 represents an important virtue of stereotactic thermal ablation, leading to enhanced safety (60, 62). It further allows to safely target a multitude of tumors in one treatment session (average number of tumor targeted in included studies up to 6; **Table 2**), keeping oncological indications for ablation treatment in mind. The high efficiency in tumor targeting did not unanimously translate into reduced overall procedure durations, where confidence intervals were wide and conflicting results were reported in comparison to free-hand targeting. An increased complexity in hardware setup and procedural workflows has been highlighted as potential drawbacks when using such novel technology (40). One way to optimize procedural efficiency when applying such novel technology is to invest in initial training of staff including anesthesia to create smooth and standardized clinical workflows in multidisciplinary teams. The latter allow relatively steep learning curves (36) and a reduction of inter-operator variance as a factor influencing outcomes after thermal ablation (47). The present study confirms enhanced reliability and reproducibility of outcomes as being one of the key advantages of using stereotactic ablation techniques (50).

Overall reported treatment efficacy rates were encouraging, leading to increased early local tumor control when using stereotactic navigation as opposed to manual guidance as shown in the latest available comparative studies (**Figure 7**). Of importance is the inconsistency in reported definitions describing technique efficacy and LTP (**Figure 5**). While standardized terminology and reporting of outcomes criteria after image-guided ablation have been proposed (24), definitions allow wide ranges of author-dependent interpretations. Especially the definition of “secondary treatment efficacy” allows for differing and unspecified numbers of retreatments over an undefined follow-up period to be included. Consequently, detectable tumor at the ablation site might only be defined as “local tumor progression” rates at 6 or 12 months after the initial ablation. This renders a straightforward comparison of reported results impossible. Despite subgroup meta-analysis according to time point of follow-up, subgroups remained highly heterogeneous due to variations in included patients, lesions, and applied types of stereotactic ablative treatments. This highlights the need for further standardization of follow-up definitions toward more clearly defined ranges of follow-up time points and more precise terminology. Using novel segmentation technology, a quantitative volumetric assessment of ablation margins will in the future allow a quantified distinction between “incomplete ablation” and true “tumour recurrence/progression at the ablation site” (65). Integrating such precise assessment of treatment success into refined follow-up terminology could contribute to the generation of more reproducible and comparable outcomes after stereotactic thermal ablation.

Varying types of navigation modalities were applied in the included studies, such as optically tracked aiming devices or EM-tracked ablation probes combined with US to CT/MR image fusion. An upcoming group of navigation systems uses robotic assistance devices, relying on mechanical tracking for automatic orientation of the robotic arm but still requiring manual insertion of ablation probes. These comprise important but only initial steps toward automation and standardization of stereotactic ablation on a larger scale. Future generations of robotic devices aim to perform automated probe positioning according to defined trajectories (66) and generate individualized ablation volumes for specified tumor configurations (67). The development of automated and dynamic patient tracking will further allow to address current challenges related to motion artifacts and facilitate ablation under sedation. No clear benefit regarding targeting accuracy when using current robotic vs. non-robotic approaches can be confirmed to date.

A potential limitation of the present work involves a potential bias in interpretation of qualitative analyses due to overlapping cohorts (**Figure 1**), which was not feasible to address, since studies overlapped varyingly for different outcomes assessed. Another important issue is the heterogeneity in tumor entities and applied ablation systems in the included studies, since variability in the response to applied ablation energies across tumor types and devices is known (68, 69). In this study, we deliberately focused on targeting accuracy, procedural efficiency, and primary technique efficacy, which were not expected to differ significantly between tumor types, since they are primarily related to procedural technique rather than oncological aspects. Contrarily, many tumor-specific determinants affect ablation site recurrences and long-term tumor control, such as satellite nodules detectable on pathology but not imaging, underlying liver disease, mutational status and location of primary tumor, and different types and time points of chemotherapy regimens (70, 71). Lastly, combination therapies with other interventional treatments complicate comparisons of long-term oncological outcomes. A variability in the calculation of morbidity and mortality rates (assessment per patient vs. per intervention vs. per lesion, 30- vs. 90-day period) remained. In some studies, it was further unclear if the positioned ablation probes, for which targeting accuracies were reported, were the ones applied for subsequent thermal ablation. This might affect conclusions regarding a relationship of targeting accuracy with resulting treatment efficacy. While accurate tumor targeting is the initial step for successful ablation, an independent correlation with early ablation site recurrence has been described (29).

As technology evolves, it can be expected that stereotactic and robotic interventions will increasingly become an integral part of the multimodality management of malignant liver tumors. Due to the increasing expertise in specialized centers, treatment indications for stereotactic thermal ablation are increasingly expanded. The present summary shows the encouraging results in all reported outcomes including for patients with tumors in difficult intrahepatic locations [centrally, liver dome (31), segment 1 (53)], very large tumors (49), and “vanishing” or CT-invisible tumors using image fusion (72). In practice, minimally invasive stereotactic ablation approaches are beneficial especially in these situations, where they allow a curative intended treatment when conventional image guidance techniques preclude an efficient and safe targeting. Key challenges remaining to be addressed when using current navigation technology are potential inaccuracies due to motion artifacts (73) and concerns regarding cost effectiveness (74).

## Conclusions

Advances in stereotactic navigation technology allow highly precise, safe, and efficient minimally invasive ablation of malignant liver tumors, potentially leading to enhanced early treatment efficacy compared to traditional guidance techniques. Heterogeneity in terminology and time points in follow-up assessment limits comparability of safety and treatment efficacy among studies, highlighting the crucial need for further standardization and guidelines for follow-up definitions.

## Data Availability Statement

The original contributions presented in the study are included in the article/**Supplementary Material**. Further inquiries can be directed to the corresponding author.

## Author Contributions

PT and IP were involved in study concept and design, data acquisition, data analysis and interpretation, statistical analysis, and manuscript preparation and editing. SR and JE were involved in study concept and design, data analysis and interpretation, and manuscript editing and review. SW, KJ, DC, and JF were involved in study concept and design and manuscript editing and review. All authors contributed to the article and approved the submitted version.

## Funding

PT reports funding from the Prof. Dr. Max Cloëtta Foundation, Switzerland, and from the Swiss Cancer League (BIL KLS-4894-08-2019). JE was supported by Region Stockholm (clinical postdoctoral appointment) and funded by Ruth and Richard Julin Foundation. Sources of funding had no involvement in study design, data collection/analysis, or manuscript preparation.

## Conflict of Interest

The authors declare that the research was conducted in the absence of any commercial or financial relationships that could be construed as a potential conflict of interest.

## Publisher’s Note

All claims expressed in this article are solely those of the authors and do not necessarily represent those of their affiliated organizations, or those of the publisher, the editors and the reviewers. Any product that may be evaluated in this article, or claim that may be made by its manufacturer, is not guaranteed or endorsed by the publisher.

## Supplementary Material

Supplementary File 1 PRISMA checklist

Supplementary File 2 All data extracted for systematic review and meta-analysis

Supplementary File 3 Meta-Analysis results including sensitivity analyses and risk of bias assessment
