# Supplementary material for: Stereotactic and Robotic Minimally Invasive Thermal Ablation of Malignant Liver Tumors: A Systematic Review and Meta-Analysis
Source: Front Oncol. 2021 Sep 23;11:713685. doi: 10.3389/fonc.2021.713685 (PMC8495244; doi:10.3389/fonc.2021.713685)
Supplement: Supplementary File 3 — Meta-Analysis results including sensitivity analyses and risk of bias assessment [file DataSheet_1.docx]

**Meta-Analysis Results**

**Lateral Errors**

***Random effects***

**Weights**

study names weights

Tinguely 2020 : 29.515%

Heerink 2019 : 9.760%

BeyerA 2018 : 24.848%

Engstrand 2016: 18.775%

WidmannB 2011 : 17.102%

**Summary**

Continuous Random-Effects Model

Metric:

Model Results

Estimate Lower bound Upper bound Std. error p-Value

3.720 3.041 4.399 0.346 < 0.001

Heterogeneity

tau^2 Q(df=4) Het. p-Value I^2

0.389 16.226 0.003 75.347

**Forest Plot**

***Fixed effects inverse variance***

**Weights**

study names weights

Tinguely 2020 : 74.926%

Heerink 2019 : 1.568%

BeyerA 2018 : 14.025%

Engstrand 2016: 5.267%

WidmannB 2011 : 4.214%

**Summary**

Continuous Fixed-Effect Model

Metric:

Model Results

Estimate Lower bound Upper bound Std. error p-Value

3.478 3.253 3.703 0.115 < 0.001

Heterogeneity

Q(df=4) Het. p-Value

16.226 0.003

**Forest Plot**

**Angular Errors**

***Random effects***

**Weights**

study names weights

Tinguely 2020 : 27.196%

Heerink 2019 : 12.768%

BeyerA 2018 : 21.224%

Engstrand 2016: 15.312%

WidmannB 2011 : 23.499%

**Summary**

Continuous Random-Effects Model

Metric:

Model Results

Estimate Lower bound Upper bound Std. error p-Value

2.410 1.742 3.079 0.341 < 0.001

Heterogeneity

tau^2 Q(df=4) Het. p-Value I^2

0.422 24.959 < 0.001 83.974

**Forest Plot**

***Fixed effects inverse variance***

**Weights**

study names weights

Tinguely 2020 : 88.594%

Heerink 2019 : 0.869%

BeyerA 2018 : 3.391%

Engstrand 2016: 1.260%

WidmannB 2011 : 5.887%

**Summary**

Continuous Fixed-Effect Model

Metric:

Model Results

Estimate Lower bound Upper bound Std. error p-Value

2.013 1.885 2.141 0.065 < 0.001

Heterogeneity

Q(df=4) Het. p-Value

24.959 < 0.001

**Forest Plot**

**Euclidean Errors**

***Random effects***

**Weights**

study names weights

Heerink : 14.838%

BeyerA : 22.251%

Engstrand: 19.581%

BeyerB : 21.334%

Mbaliske : 21.996%

**Summary**

Continuous Random-Effects Model

Metric:

Model Results

Estimate Lower bound Upper bound Std. error p-Value

5.300 3.862 6.737 0.733 < 0.001

Heterogeneity

tau^2 Q(df=4) Het. p-Value I^2

2.337 53.723 < 0.001 92.554

**Forest Plot**

***Fixed effects inverse variance***

**Weights**

study names weights

Heerink : 2.553%

BeyerA : 41.095%

Engstrand: 8.026%

BeyerB : 17.885%

Mbaliske : 30.441%

**Summary**

Continuous Fixed-Effect Model

Metric:

Model Results

Estimate Lower bound Upper bound Std. error p-Value

4.414 4.059 4.770 0.181 < 0.001

Heterogeneity

Q(df=4) Het. p-Value

53.723 < 0.001

**Forest Plot**

**Overall Complications**

***Random effects – Subgroup analysis***

**Subgroup Summary**

Binary Random-Effects Model

Metric: Proportion

Model Results

Subgroups Studies Estimate Lower bound Upper bound Std. error p-Val z-Val

Subgroup 1 3 0.342 0.015 0.668 0.167 0.040 2.053

Subgroup 2 2 0.028 -0.024 0.080 0.027 0.295 1.047

Subgroup 3 11 0.091 0.038 0.144 0.027 < 0.001 3.377

Overall 16 0.114 0.067 0.161 0.024 < 0.001 4.717

Heterogeneity

Studies Q (df) Het. p-Val I^2

Subgroup 1 55.716 (2) < 0.001 9641 %

Subgroup 2 1.290 (1) 0.256 2251 %

Subgroup 3 60.733 (10) < 0.001 8353 %

Overall 123.961 (15) < 0.001 8790 %

**Forest Plot**

***Fixed effects inverse variance – Subgroup analysis***

**Subgroup Summa**

Binary Fixed-effect Model - Inverse Variance

Metric: Proportion

Model Results

Subgroups Studies Estimate Lower bound Upper bound Std. error p-Val z-Val

Subgroup 1 3 0.083 0.052 0.113 0.015 < 0.001 5.367

Subgroup 2 2 0.022 -0.015 0.059 0.019 0.238 1.179

Subgroup 3 11 0.061 0.043 0.080 0.010 < 0.001 6.424

Overall 16 0.060 0.046 0.075 0.007 < 0.001 8.077

Heterogeneity

Studies Q (df) Het. p-Val I^2

Subgroup 1 55.716 (2) < 0.001 0 %

Subgroup 2 1.290 (1) 0.256 0 %

Subgroup 3 60.733 (10) < 0.001 0 %

Overall 123.961 (15) < 0.001 0 %

**Forest Plot**

**Major Complications**

***Random effects - Subgroup analysis***

**Subgroup Summary**

Binary Random-Effects Model

Metric: Arcsine of Square Root Proportion

Model Results

Subgroups Studies Estimate Lower bound Upper bound Std. error p-Val z-Val

Subgroup 1 3 0.020 0.007 0.040 0.031 < 0.001 4.593

Subgroup 2 4 0.040 0.010 0.088 0.051 < 0.001 3.983

Subgroup 3 13 0.019 0.010 0.032 0.021 < 0.001 6.681

Overall 20 0.024 0.014 0.036 0.018 < 0.001 8.638

Heterogeneity

Studies Q (df) Het. p-Val I^2

Subgroup 1 1.091 (2) 0.579 0 %

Subgroup 2 4.781 (3) 0.189 3725 %

Subgroup 3 13.133 (12) 0.359 862 %

Overall 23.962 (19) 0.198 2071 %

**Forest Plot**

***Fixed effects inverse variance - Subgroup analysis***

**Subgroup Summary**

Binary Fixed-effect Model - Inverse Variance

Metric: Arcsine of Square Root Proportion

Model Results

Subgroups Studies Estimate Lower bound Upper bound Std. error p-Val z-Val

Subgroup 1 3 0.020 0.007 0.040 0.031 < 0.001 4.593

Subgroup 2 4 0.050 0.023 0.085 0.036 < 0.001 6.190

Subgroup 3 13 0.018 0.010 0.029 0.019 < 0.001 7.205

Overall 20 0.023 0.015 0.032 0.015 < 0.001 10.314

Heterogeneity

Studies Q (df) Het. p-Val I^2

Subgroup 1 1.091 (2) 0.579 0 %

Subgroup 2 4.781 (3) 0.189 0 %

Subgroup 3 13.133 (12) 0.359 0 %

Overall 23.962 (19) 0.198 0 %

**Forest Plot**

**Mortality**

***Random effects***

**Weights**

study names weights

Tinguely : 16.452%

Schaible : 15.081%

Schullian0.5: 9.254%

Volpi : 2.399%

ZhangA : 1.799%

Heerink : 1.628%

BeyerA : 1.628%

ZhangB : 1.714%

TinguelyB : 4.713%

HirookaA : 2.399%

Engstrand : 2.228%

BeyerB : 2.999%

AbdullahA : 1.799%

Mbaliske : 2.656%

Sindram : 1.200%

Mauri : 18.509%

AbdullahB : 1.028%

WidmannA : 9.083%

WidmannB : 1.799%

Liu : 1.628%

**Summary**

Binary Random-Effects Model

Metric: Arcsine of Square Root Proportion

Model Results

Estimate Lower bound Upper bound p-Value

0.008 0.004 0.014 < 0.001

Heterogeneity

tau^2 Q(df=19) Het. p-Value I^2

0.000 7.890 0.988 0

Results (arcsine scale)

Estimate Lower bound Upper bound Std. error

0.088 0.060 0.117 0.015

**Forest Plot**

***Fixed effects inverse variance***

**Weights**

study names weights

Tinguely : 16.452%

Schaible : 15.081%

Schullian0.5: 9.254%

Volpi : 2.399%

ZhangA : 1.799%

Heerink : 1.628%

BeyerA : 1.628%

ZhangB : 1.714%

TinguelyB : 4.713%

HirookaA : 2.399%

Engstrand : 2.228%

BeyerB : 2.999%

AbdullahA : 1.799%

Mbaliske : 2.656%

Sindram : 1.200%

Mauri : 18.509%

AbdullahB : 1.028%

WidmannA : 9.083%

WidmannB : 1.799%

Liu : 1.628%

**Summary**

Binary Fixed-Effect Model - Inverse Variance

Metric: Arcsine of Square Root Proportion

Model Results

Estimate Lower bound Upper bound p-Value

0.008 0.004 0.014 < 0.001

Heterogeneity

Q(df=19) Het. p-Value

7.890 0.988

Results (arcsine scale)

Estimate Lower bound Upper bound Std. error

0.088 0.060 0.117 0.015

**Forest Plot**

**Primary Technique Efficacy**

***Random effects – Subgroup analysis***

**Subgroup Summary**

Binary Random-Effects Model

Metric: Proportion

Model Results

Subgroups Studies Estimate Lower bound Upper bound Std. error p-Val z-Val

Subgroup 1 10 0.937 0.901 0.972 0.018 < 0.001 51.950

Subgroup 2 5 0.912 0.876 0.947 0.018 < 0.001 50.236

Overall 15 0.925 0.895 0.955 0.015 < 0.001 60.311

Heterogeneity

Studies Q (df) Het. p-Val I^2

Subgroup 1 33.122 (9) < 0.001 7283 %

Subgroup 2 11.235 (4) 0.024 6440 %

Overall 79.327 (14) < 0.001 8235 %

**Forest Plot**

***Fixed effects inverse variance – Subgroup analysis***

**Subgroup Summary**

Binary Fixed-effect Model - Inverse Variance

Metric: Proportion

Model Results

Subgroups Studies Estimate Lower bound Upper bound Std. error p-Val z-Val

Subgroup 1 10 0.975 0.966 0.984 0.005 < 0.001 211.124

Subgroup 2 5 0.911 0.892 0.930 0.010 < 0.001 93.519

Overall 15 0.963 0.955 0.971 0.004 < 0.001 230.833

Heterogeneity

Studies Q (df) Het. p-Val I^2

Subgroup 1 33.122 (9) < 0.001 0 %

Subgroup 2 11.235 (4) 0.024 0 %

Overall 79.327 (14) < 0.001 0 %

**Forest Plot**

**Primary Technique Efficacy Comparative**

***Random effects***

**Weights**

study names weights

Schaible: 75.843%

Zhang : 6.744%

Heerink : 4.449%

BeyerA : 4.398%

ZhangB : 4.467%

BeyerB : 4.098%

**Summary**

Binary Random-Effects Model

Metric: Odds Ratio

Model Results

Estimate Lower bound Upper bound p-Value

1.944 1.183 3.193 0.009

Heterogeneity

tau^2 Q(df=5) Het. p-Value I^2

0.000 4.121 0.532 0

Results (log scale)

Estimate Lower bound Upper bound Std. error

0.665 0.168 1.161 0.253

**Forest Plot**

***Fixed effects Mantel Haenszel***

**Weights**

study names weights

Schaible: 64.432%

Zhang : 7.126%

Heerink : 12.710%

BeyerA : 3.619%

ZhangB : 4.242%

BeyerB : 7.871%

**Summary**

Binary Fixed-Effect Model - Mantel Haenszel

Metric: Odds Ratio

Model Results

Estimate Lower bound Upper bound p-Value

1.879 1.155 3.056 0.011

Heterogeneity

Q(df=5) Het. p-Value

4.139 0.530

Results (log scale)

Estimate Lower bound Upper bound Std. error

0.631 0.144 1.117 0.248

**Forest Plot**

***Risk of Bias Assessment***

library(meta)

library(tidyverse)

library(readxl)

**Load Data**

eff.comp <- read_excel("EfficacyComparative_RBiostat.xlsx")

colnames(eff.comp) <- c("Study", "Event.A", "Total.A", "Event.B", "Total.B")

head(eff.comp)

## # A tibble: 6 x 5

## Study Event.A Total.A Event.B Total.B

## <chr> <dbl> <dbl> <dbl> <dbl>

## 1 Schaible 20 219 249 91 119

## 2 Zhang 19 18 20 16 19

## 3 Heerink 19 17 20 20 21

## 4 BeyerA 18 17 18 15 18

## 5 ZhangB 18 18 19 21 24

## 6 BeyerB 17 32 34 29 30

**Funnel Plot**

m.eff.comp <- metabin(Event.A, Total.A, Event.B, Total.B,

data = eff.comp, studlab =

Study, sm = "OR", backtransf = FALSE)

funnel.meta(m.eff.comp, studlab = TRUE)

**Egger test**

K.min = 1, otherwise metabias complains about not having enough samples (n=10)

metabias(m.eff.comp, method.bias = "linreg", k.min = 1)

## Linear regression test of funnel plot asymmetry

##

## Test result: t = -1.24, df = 4, p-value = 0.2840

##

## Sample estimates:

## bias se.bias intercept se.intercept

## -0.7298 0.5904 1.0278 0.3663

##

## Details:

## - multiplicative residual heterogeneity variance (tau^2 = 0.7455)

## - predictor: standard error

## - weight: inverse variance

## - reference: Egger et al. (1997), BMJ
